# Molecular screening for rickettsial bacteria and piroplasms in ixodid ticks surveyed from white-tailed deer (*Odocoileus virginianus*) and nilgai antelope (*Boselaphus tragocamelus*) in southern Texas^☆^

**DOI:** 10.1016/j.ijppaw.2020.11.002

**Published:** 2020-11-17

**Authors:** Pia U. Olafson, Beverly Greta Buckmeier, Melinda A. May, Donald B. Thomas

**Affiliations:** a USDA-ARS Knipling-Bushland Livestock Insects Research Laboratory, 2700 Fredericksburg Rd., Kerrville, TX, 78028, USA; b USDA-ARS Cattle Fever Tick Research Laboratory, 22675 N. Moorefield Road, Moore Air Base, Edinburg, TX, 78541, USA

**Keywords:** Anocenter nitens, Horse tick, Amblyomma maculatum, Amblyomma inornatum, Rickettsia parkeri, Theileria cervi, Rickettsia amblyommatis

## Abstract

A survey of ixodid ticks parasitizing white-tailed deer (*Odocoileus virginianus*) and nilgai antelope (*Boselaphus tragocamelus*) was completed during the 2018–2019 public hunt season on the Laguna Atascosa National Wildlife Refuge (Cameron County, Texas) and the East Foundation's El Sauz Ranch in nearby Willacy County (Texas). *Anocenter nitens* was the predominant tick species identified with 5% of these ticks collected from nilgai. All life stages were encountered in high numbers on white-tailed deer, indicating that deer may be a primary host in this region. *Amblyomma maculatum* and *Amblyomma inornatum* were identified from both hosts, while *Ixodes scapularis* was encountered only on white-tailed deer. This is the first published record of *A. inornatum* on nilgai. A subset of ticks was used in PCR assays to detect *Rickettsia* spp., family *Anaplasmataceae*, *Borrelia* spp., and *Theileria-Babesia* spp. *Borrelia* spp. were not detected in any of the ticks analyzed. *Rickettsia parkeri* was detected in three *A. maculatum* adult ticks from deer, *Rickettsia* sp. endosymbiont sequences were present in all *I. scapularis* ticks, and *Rickettsia amblyommatis* was detected in three *A. inornatum* adult ticks from deer. Sequence analysis of *Anaplasmataceae-*positive amplicons from *A. nitens* and *A. maculatum* had low percent identity to published *Anaplasma* spp. sequences, suggesting a unique *Anaplasma* sp. may be circulating in the population. *Anaplasma platys* was detected from *A. nitens* larvae and an *Ehrlichia* sp. Delta strain was present in *A. maculatum*, both of unknown pathogenicity towards deer. *Theileria cervi* was detected in all stages of *A. nitens* ticks, and positive ticks originated from 27 of 31 deer and a single nilgai sampled from throughout the survey site. The primary vector for *T. cervi* is absent from this region, suggesting *T. cervi* is possibly maintained by a different tick species.

## Highlights

• *Anocenter nitens* predominates on white-tailed deer and nilgai in southern Texas.
• *Theileria cervi* was detected in *Anocenter nitens* ticks from deer and a single nilgai.
• Three *Theileria cervi* genotypes were detected in this region of southern Texas.
• Unique *Anaplasma* sp. detected in *A. nitens*; low identity to known *Anaplasma* sp.
• *Amblyomma inornatum* ticks were identified on nilgai; a first record on this host.

## Graphical abstract

**Figure:**
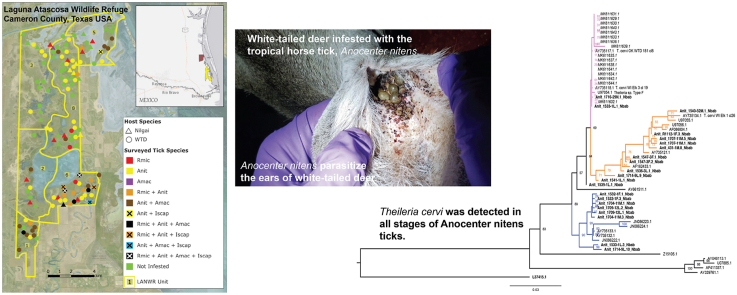

## 1 Introduction

The border region of south Texas shares a tropical tick fauna with Mexico that is unlike that found in the rest of the USA. Cattle fever ticks (*Rhipicephalus* (*Boophilus*) spp.), vectors of bovine babesiosis, are prevalent in Mexico and found intermittently in the USA only in the counties bordering the Rio Grande (Lohmeyer et al., 2011). Beginning in 2014 however, a population became established on sylvatic hosts in the coastal areas of Cameron and Willacy counties (Texas) (Lohmeyer et al., 2018; Olafson et al., 2018). While cattle fever ticks are one-host ticks that use bovines as their preferred hosts, white-tailed deer (*Odocoileus virginianus* Zimmerman; hereafter referred to as deer) have long been known as secondary hosts (Pound et al., 2010), and evidence suggests that deer are neither susceptible to infection nor serve as reservoirs of *Babesia bovis*, the causative agent of bovine babesiosis (Ueti et al., 2015). Nilgai antelope (*Boselaphus tragocamelus* Pallas; hereafter referred to as nilgai)*,* are even more concerning as they are competent hosts of the tick vector and are vehicles for tick dissemination and, as a bovine, are potential reservoirs of bovine babesiosis (Foley et al., 2017; Lohmeyer et al., 2018). The dynamics of cattle fever tick populations relative to other tick fauna that occupy this landscape is understudied, as are the parasites that may be harbored by these tick populations. Surveys of ticks on wild pigs (*Sus scrofa*), deer, and nilgai identified a variety of tick species that share this environment, including *Amblyomma maculatum* Koch*, Amblyomma mixtum* Koch, *Amblyomma tenellum* Koch, *Ixodes scapularis* Say, *Dermacentor variabilis* Say, and *Dermacentor halli* McIntosh (Corn et al., 2016; Feria-Arroyo et al., 2014; Olafson et al., 2018; Sanders et al., 2011), but molecular surveillance for parasites is limited.

For this study, deer and nilgai antelope were sampled for tick infestations at the Laguna Atascosa National Wildlife Refuge (LANWR) in Cameron County, Texas and on the East Foundation's El Sauz Ranch in Willacy County, Texas. A subsample of these tick specimens was then screened for bacteria and piroplasms of concern to human and animal health.

## 2 Materials and methods

### 2.1 Study site and tick collections

**Fig. 1:**
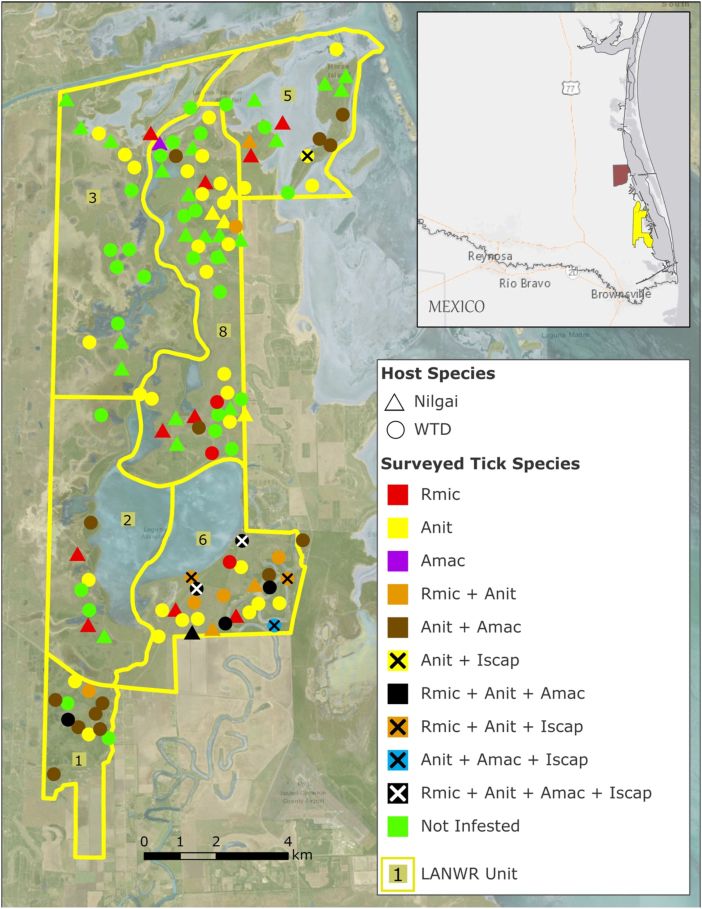
Prevalence of ixodid ticks on white-tailed deer and nilgai on the Laguna Atascosa Wildlife Refuge (LANWR) in Cameron County (Texas). The LANWR is divided into Units, outlined in yellow. Deer and nilgai were harvested from the indicated units during the public hunt season in 2018–2019. Deer are identified by circles, nilgai are identified by triangles. Ixodid tick species collected either singly or in combination from these hosts are indicated by a different fill color presented in the legend. Animals from which no ticks were collected are shaded in green (‘Not infested’). Rmic: *Rhipicephalus* (*Boophilus*) *microplus*; Anit: *Anocenter nitens*; Amac: *Amblyomma maculatum*; Iscap: *Ixodes scapularis*. **Inset**: The LANWR units are shaded in yellow, while the East Foundation's El Sauz Ranch located north of the LANWR is shaded in maroon.

Deer and nilgai were censused for tick infestations at the Laguna Atascosa National Wildlife Refuge (LANWR; Cameron County, Texas). Animals were harvested from Units 1, 2, 3, 5, 6, and 8 as part of the open public hunts (Fig. 1). Deer and nilgai were also inspected at East Foundation's El Sauz Ranch (ESR) located approx. 35 km north of LANWR Units 3 and 5. This combined census effort was part of surveillance for invasive cattle fever ticks, and harvested (LANWR) or live-captured (ESR) animals were scratch-inspected for ticks by inspectors from the US Department of Agriculture (USDA)-Animal Plant Health Inspection Service-Veterinary Services-Cattle Fever Tick Eradication Program and the Texas Animal Health Commission. If harvested, carcasses were typically field dressed, could be many hours post-mortem, and had been transported to the check station from the harvest site. If live-captured, animals were scratch-inspected for ticks prior to release. The unit within the LANWR where each animal was taken was recorded, and the position was based on hunter recall. Ticks collected from each individual host were placed in a glass vial, and the vial was marked with a unique identifier for the deer or nilgai. Vials of ticks were delivered to the USDA-Agricultural Research Service, Cattle Fever Tick Research Lab (Edinburg, Texas) where ticks per individual host were enumerated and identified to species using taxonomic keys (Estrada-Pena et al., 2005; Guzmán-Cornejo et al., 2011; Keirans and Clifford, 1978; Nava et al., 2014; Yunker et al., 1986). The ticks were subsequently archived in absolute ethanol and stored at room temperature.

### 2.2 Tick specimens for molecular analysis

Genomic DNA (gDNA) was isolated from a sub-sample of ticks collected from the deer and nilgai described in Section 2.1. *Anocenter nitens* Neumann gDNA was isolated from adults (N = 235; 84 female, 151 male), nymphs (N = 115) and 55 larval samples with 1–15 larvae comprising each sample. These were collected from 31 deer hosts, while *A. nitens* gDNA was also isolated from adults (N = 70; 32 female, 38 male), nymphs (N = 12) and two larval samples collected from five nilgai hosts. All *A. nitens* ticks were collected from hosts in the LANWR. gDNA was isolated from *A. maculatum* collected from seven deer hosts in the LANWR (N = 16; 3 females, 12 males, 1 nymph) and four deer live-captured at the ESR (N = 17; 13 females, 4 males)., gDNA was isolated from *Ixodes scapularis* Say adults obtained from six deer hosts in the LANWR (N = 29; 23 females, 6 males) and live-captured deer on ESR (N = 8 ticks, all female, of 52 total deer inspected). *Amblyomma inornatum* Banks adults obtained from three deer hosts in the LANWR (N = 3 ticks; 1 female, 2 males) were used for gDNA isolation. Ethanol was decanted from individual vials housing the tick samples. The ticks were then rinsed in three changes of Tris-EDTA, pH 8.0, dried on paper towels, and placed in Petri dishes. All specimens were dried under a chemical fume hood for 5 h, after which each adult or nymph was placed into a Kimble® DNAse/RNase-free, 1.5 ml reaction tube. Dried *A. nitens* larvae were either isolated individually or pooled into a total of 55 larval samples. Each tube was chilled in liquid nitrogen, and the tick or larval pool was macerated using an individual, liquid nitrogen-cooled, disposable Kimble® pestle. Tick gDNA was isolated using the DNEasy® Blood and Tissue Kit (Qiagen, Germantown, MD), and gDNAs were eluted from the column in 100 μl Tris HCl, pH 8.0. Samples were stored at −20 °C until further analysis.

### 2.3 Tick-borne bacteria and piroplasm detection by PCR

Tick gDNA samples were tested for presence of *Rickettsia* spp., family *Anaplasmataceae*, *Borrelia* spp., and *Theileria-Babesia* spp. DNA using PCR assays to target the *Rickettsia* surface cell antigen, sca0 (*rompA*) (Regnery et al., 1991), family *Anaplasmataceae* heat shock protein *groEL* (Tabara et al., 2007), *Theileria-Babesia* 18S SSU rRNA spanning the V4 region (Oosthuizen et al., 2008; Ueti et al., 2015), and *Borrelia* flagellin *flaB* (Barbour et al., 1996). Oligonucleotide primer sequences, reaction conditions, and cycling parameters are summarized in the supplementary file (Table S1). Assays were conducted in a 20 μl reaction volume with Platinum™ *Taq* DNA Polymerase (ThermoFisher Scientific, Waltham MA). *Anocenter nitens* adult, nymph, and larval sample gDNAs were initially screened in pools. Each pool was comprised of 5 μl tick gDNA from up to ten individuals/larval samples in a total volume of 50 μl. An aliquot of the pooled gDNA (5 μl) was used as template in an initial screen for parasite detection. If a positive amplicon was detected, individual tick or larval sample gDNAs that comprised the pool were further screened to assess the number of positive ticks or larval samples (2 μl gDNA in a 20 μl reaction volume). *Amblyomma maculatum, A. inornatum*, and *I. scapularis* ticks were screened individually using a 2 μl gDNA aliquot in a 20 μl reaction volume.

Positive control DNAs were included in all diagnostic PCR assay runs. Template gDNA was procured from the American Type Culture Collection (Manassas, VA) for *Rickettsia rickettsii* (NR-48826) and *Borrelia burgdorferi* (35210D-5.) Also used were template from an *Ehrlichia chaffeensis*-positive *Amblyomma americanum* adult female tick (EC-16-TN; 2 μl) kindly provided by Dr. Rebecca Trout Fryxell (University of Tennessee, Knoxville) and gDNA isolated from whole blood of a *Theileria cervi*-infected white-tailed deer doe. A no-template, water control was included with each diagnostic PCR assay run.

Amplicons were prepared for sequencing using the DNA Clean-and-Concentrator kit (Zymo Research), and these samples were submitted to Genscript (Piscataway, NJ) for bidirectional Sanger sequencing. Given the number of family *Anaplasmataceae-* and *Theileria-Babesia*-positive *A. nitens* ticks, at least one individual tick amplicon per host animal was sequenced in full length to validate identity. A larger, *Theileria* 18S rRNA SSU fragment (1660–1665 bp) was amplified from selected *A. nitens* ticks to obtain further information about the isolate types circulating in this population. A hemi-nested approach was used to amplify the fragment from individual ticks, and primer sequences, reaction conditions, and cycling parameters are summarized in the supplementary file (Table S1). Assays were conducted in a 20 μl reaction volume with 50× Advantage® High Fidelity 2 Polymerase (Takara, Mountain View CA). A number of these amplicons were polymorphic at numerous positions, thus were ligated to the pCR™4-TOPO™ plasmid vector (ThermoFisher Scientific), cloned in One Shot™ TOP10 Chemically Competent *E. coli* (ThermoFisher Scientific), and the resulting plasmids of at least three clones were sequenced using multiple primers to span the full length of the clone (Table S1). All sequences were submitted to the GenBank repository. These include: the smaller *T. cervi* 18S SSU rRNA amplicons from LANWR *A. nitens* ticks (MW008539-MW008550) and the larger (1660–1665 bp) 18S SSU rRNA cloned fragments from LANWR *A. nitens* ticks (MW008518-MW008538). Also submitted were: *A. nitens groEL* amplicons from adult (MW008554) and larval (MW0 08555) templates, *A. maculatum groEL* from ESR adults (MW008557-MW008558) and a LANWR adult (MW008556), *A. inornatum* sca0 amplicon from LANWR adults (MW241133), *I. scapularis* sca0 amplicons from adults (MW008552) and the *A. maculatum* sca0 amplicon from LANWR and ESR adults (MW008551).

### 2.4 Phylogenetic analysis

Nucleotide sequences from representative *Theileria* and *Babesia* 18S SSU rRNAs were obtained from GenBank (publicly available database) and aligned with *Theileria* sequences (1660–1665 bp) from *A. nitens* using the MUSCLE algorithm (Edgar, 2004). Similarly, representative sequences annotated as rickettsial endosymbionts of *Ixodes* spp. and *Amblyomma* spp. were obtained and aligned with the *Rickettsia* sp. sequences from *I. scapularis* and *A. maculatum* obtained in this study. Alignments were used to construct a maximum likelihood phylogeny with the web server version of IQ-TREE software (Trifinopoulos et al., 2016) with best-fit substitution model (Kalyaanamoorthy et al., 2017) and branch support assessed with 10,000 replicates of UFBoot bootstrap approximation (Hoang et al., 2018). The *Theileria* 18S SSU rRNA phylogenetic tree was rooted using *Toxoplasma gondii* (L37415) as an outgroup, as it belongs to a different class of apicomplexa. The *Rickettsia* spp. tree was rooted at midpoint.

## 3 Results

### 3.1 Prevalence of ixodid ticks on white-tailed deer and nilgai in southern Texas

A total of 90 deer and 42 nilgai were harvested as part of the 2018-19 public hunts on the LANWR. The location within the LANWR where deer and nilgai were harvested, along with color coding for the tick species identified per host is presented in Fig. 1. A summary of the number of hosts on which various tick species were identified either singly or in combination with other tick species is presented in Table 1, Table 2, respectively. There were 27 deer and 22 nilgai from which no ticks were found (‘Not Infested’, Fig. 1). Thirty-five deer were infested with one species of tick, while 28 deer were infested with between two and four species of ticks. Fifteen nilgai were infested with one species of tick, and four nilgai were infested with two or three species of ticks.

**Table 1:** Numbers of white-tailed deer and nilgai hosts from the Laguna Atascosa National Wildlife Refuge infested with one species of tick. Numbers of female and male hosts found infested per tick species are listed. Also listed are the number of hosts from which no ticks were identified ('Not Infested').

					Species of tick identified Number of female (F) or male (M) hosts found infested with tick species									
Host	Total Hosts		Hosts Not Infested		*Rhipicephalus microplus*		*Anocenter nitens*		*Amblyomma* *maculatum*		*Amblyomma inornatum*		*Ixodes scapularis*	
	F	M	F	M	F	M	F	M	F	M	F	M	F	M
Nilgai	24	13	15	7	5	5	3	1	1	0	0	0	0	0
Deer	26r	36	11	16	0	3	15	17	0	0	1	0	0	0

**Table 2:** Records of white-tailed deer and nilgai hosts from the Laguna Atascosa Wildlife Refuge infested with multiple species of ticks.

Host	Total		Rm/An/Am^a^		Rm/An^a^		An/Is^a^		An/Am^a^		Rm/An/Is^a^		Rm/An/Am/Is^a^			An/Am/Is^a^		
	F	M	F	M	F	M	F	M	F	M	F	M	F	M	F		M	
Nilgai	1	3	1	0	0	3	0	0	0	0	0	0	0	0	0		0	
Deer	4	24	1	2	1	4	0	1	2	12	0	2	0	2	0		1	

a Rm: *Rhipicephalus microplus*; An: *Anocenter nitens*; Am: *Amblyomma maculatum*; Is: *Ixodes scapularis*.

**Fig. 2:**
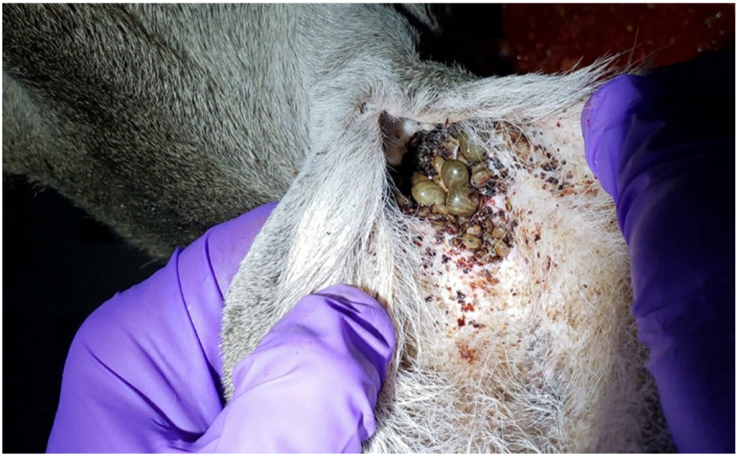
*Anocenter nitens*, the tropical horse tick, infesting a white-tailed deer. As in horses, *A. nitens* ticks preferentially infest the ears of this cervid host. Photo Credit: Emma Mitchell

The predominant tick species collected from these hosts was *A. nitens* with a total of 3129 ticks, comprising 275 adult females, 435 adult males, 386 nymphs, and 2033 larvae. Of these, 39 females, 50 males, 24 nymphs, and 50 larvae were collected from eight of the nilgai. These animals were harvested from all public hunt Units of the LANWR and, as in horses, the preferred site of *A. nitens* infestation on deer was in the ears (Fig. 2). *Rhipicephalus* (*B.*) *microplus* Canestrini ticks (N = 285) were also identified from both hosts in the LANWR and comprised 71 adult females, 84 adult males, and 130 nymphs. These hosts were harvested from Units 1, 2, 5, 6, and 8 (Fig. 1). A total of 47 *A. maculatum* ticks comprising 19 adult females, 24 adult males, and 2 nymphs were collected from 19 white-tailed deer, while one adult female and one adult male were collected separately from two nilgai. These hosts were harvested from Units 1, 2, 5, 6, and 8 (Fig. 1). Thirty-one female and six male *I. scapularis* ticks were collected from deer in Units 5 and 6 and none were identified from nilgai hosts (Fig. 1). Two adult female and two adult male *A. inornatum* ticks were identified on three deer and one nilgai from Units 1, 5, 6, and 8 of the LANWR.

### 3.2 Rickettsial and piroplasm DNA detected in ixodid ticks collected from white-tailed deer and nilgai in southern Texas

Results of molecular screening assays are summarized in Table 3. *Borrelia* sp. was not detected in any of the ticks sampled. All *A. nitens* ticks were negative for *Rickettsia* sp. Approximately 21% of *A. nitens* samples were *Anaplasmataceae*-positive (N = 103/489) and represent ticks collected from 17 deer hosts in the LANWR. The proportion of *Anaplasmataceae*-positive *A. nitens* were similar between adult females (0.39) and males (0.40) with a lower proportion of positives detected from nymphs (0.15) and larval samples (0.07). Sequence data was obtained for at least one individual tick amplicon per host animal. On one of the hosts, 39 of 57 ticks were *Anaplasmataceae*-positive. Five representative sequences were obtained from this host for a total of 22 *Anaplasmataceae*-positive amplicon sequences. Seventeen of these sequences, all from adult ticks, were 100% identical to one another and displayed 81–82% nucleotide sequence identity to *Anaplasma bovis* and *Anaplasma phagocytophylum groEL* sequences in GenBank (e.g., GenBank MH255900, MH255905). The remaining five amplicons were 100% identical to one another and 99% identical to *Anaplasma platys groEL* isolates (e.g., GenBank MN202021), all amplicons of which were from larval templates.

**Table 3:** Detection of tick-borne parasites harbored by ixodid ticks collected from white-tailed deer and nilgai hosts in south Texas.

Tick Species (Host)	*Anaplasmatacae* sp. *groEL* positive				Rickettsia sp. rompA positive				Theileria-Babesia sp. 18S SSU rRNA positive				
	Female	Male	Nymphs	Larval Pools	Female	Male	Nymphs	Larval Pools	Female		Male	Nymphs	Larval Pools
	# positive/ total number (proportion positive of total)				# positive/ total number (proportion positive of total)				# positive/ total number (proportion positive of total)				
*Anocenter nitens* (White-tailed deer)	40/ 84 (0.47)	41/ 151 (0.27)	15/ 115 (0.13)	7/ 55 (0.13)	0/ 84 -	0/ 151 -	0/ 115 -	0/ 55 -	16^a^/ 84 (0.19)		60 a/ 151 (0.40)	15/ 115 (0.13)	48/ 55 (0.87)
(Nilgai)	0/ 32 -	0/ 38 -	0/ 12 -	0/ 2 -	0/ 32 -	0/ 38 -	0/ 12 -	0/ 2 -	2/ 32 (0.06)		6/ 38 (0.16)	0/ 12 -	0/ 2 -
*Amblyomma maculatum* (White-tailed deer)	6/ 16 (0.38)	2/ 16 (0.13)	0/ 1 -	n/a -	2/ 16 (0.13)	2/ 16 (0.13)	0/ 1 -	n/a -	0/ 16 -		0/ 16 -	0/ 1 -	n/a -
*Ixodes scapularis* (White-tailed deer)	0/ 31 -	0/ 6 -	n/a -	n/a -	31/ 31 (1.00)	6/ 6 (1.00)	n/a -	n/a -	0/ 31 -	0/ 6 -		n/a -	n/a -

a Of these positive *A. nitens* ticks, two adult females and six adult males were collected from a single nilgai host.

**Fig. 3:**
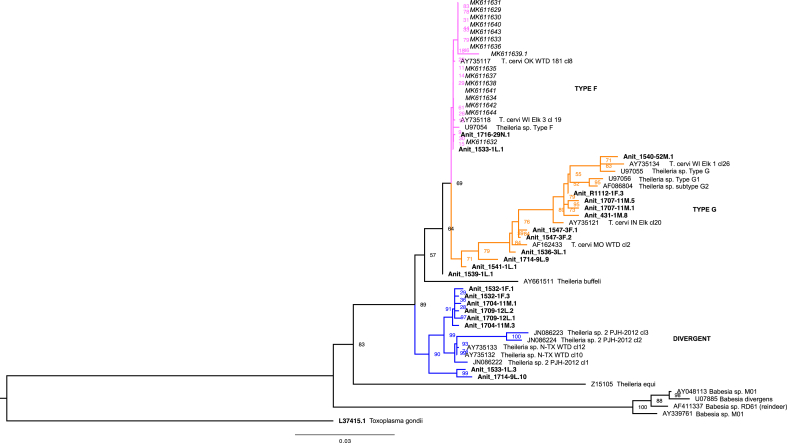
Phylogentic analysis of Theileria sp. fragments from Anocenter nitens ticks. Representative Type F, Type G, and ‘divergent’ Theileria sp. sequences were identified from individual A. nitens ticks collected from white-tailed deer and a single nilgai host (bold labels). A maximum-likelihood tree was constructed using Toxoplasma gondii as the outgroup, as it is from a different axpicomplexan class than Theileria. Branch support was assessed with 10,000 replicates of UFBoot bootstrap replication, and bootstrap percentages are indicated at each branch point in the tree. GenBank accession numbers and annotated identification for sequences used in the comparative analysis are indicated on the branch labels. Accession numbers in italics are those *T. cervi* sequences from white-tailed deer on the East Foundation's San Antonio Viejo Ranch in Starr and Jim Hogg Counties, Texas (Yu et al., 2020).

*Theileria-Babesia*-positive *A. nitens* ticks were detected in a higher proportion than *Anaplasmatacae*-positive *A. nitens* (N = 147/489, 0.30). These represented specimens collected from 27 deer and a single nilgai from the LANWR (summarized in Table S2). The highest proportion of *Theileria-Babesia*-positive ticks were from adult males (0.45) and larval samples (0.33) with similar proportions of positives detected in adult females (0.12) and nymphs (0.10). Two adult female and six adult male *A. nitens* ticks (of 24 total) were positive on the single nilgai. Sequence analyses of these smaller 430–440 bp 18S SSU rRNA fragments indicated they aligned with 95–100% nucleotide sequence identity to *Theileria cervi* and unclassified *Theileria* sp. isolates originating from cervid hosts, including white-tailed deer and elk (e.g., GenBank JN086224, U97055). These data further indicated the presence of polymorphisms, prompting isolation and sequencing of a larger, 18S SSU rRNA fragment (1660–1670 bp in size). Cloned sequences were 95–100% identical to each other and further confirmed identification of *Theileria* sp. and *T. cervi*. A phylogenetic analysis of the *A. nitens Theileria* sp. sequences and those in the public domain supported clustering of the *A. nitens* representatives with Type G and Type F *T. cervi*, as well as with a divergent *Theileria* sp. reported from white-tailed deer in north Texas (Fig. 3). Co-detection of *Anaplasma* sp. and *Theileria* sp. was observed in 38 of the *A. nitens* ticks, over half (21/38, 0.55) of which were adult males followed by eight females (0.21), three nymphs (0.08), and six larval samples (0.16).

All *A. maculatum* ticks were negative for *Theileria-Babesia* spp., while eight *A. maculatum* ticks were positive for family *Anaplasmatacae* sp. Sequence data of the amplicons for seven ticks collected from four different deer displayed 98–99% sequence identity to an *Ehrlichia* sp. Delta strain, closely related to *Ehrlichia muris* (GenBank MT681330). These four deer were live-captured and released from ESR. Data from one tick had 82% sequence identity to *An. bovis* and *An. phagocytophylum groEL* from livestock (e.g., GenBank MH255900, MH255905), and this was from a deer in the LANWR. A diagnostic amplicon for *Rickettsia* sp. sca0 (*rompA*) was detected in four adult ticks, and sequence data from three adults (1 female, 2 males) displayed 99% identity to *Rickettsia parkeri* (e.g., GenBank MG574938). Each of the *R. parkeri*-positive ticks were obtained from separate deer, two deer of which were from Unit 1 of the LANWR (Fig. 1) and one of which was from ESR. Sequence data from one female tick had 99% identity to uncultured *Rickettsia* sp. and rickettsial endosymbionts from ixodid ticks (e.g., GenBank KX077194). *Rickettsia parkeri* was co-detected with the *Ehrlichia* sp. Delta strain in one of the three *R. parkeri*-positive ticks, while the *A. bovis*-like sequence was co-detected in another *R. parkeri*-positive tick. The one rickettsial endosymbiont was co-detected with the *Ehrlichia* sp. Delta strain in a single *A. maculatum* tick.

The three *A. inornatum* ticks were negative for *Borrelia* sp., family *Anaplasmatacae*, and *Theileria-Babesia* sp., but a diagnostic amplicon for *Rickettsia* sp. sca0 was detected in two of the three ticks. Sequence data from both ticks displayed 99–100% nucleotide sequence identity to *Rickettsia amblyommatis* sca0 (e.g., GenBank MN336348).

**Fig. 4:**
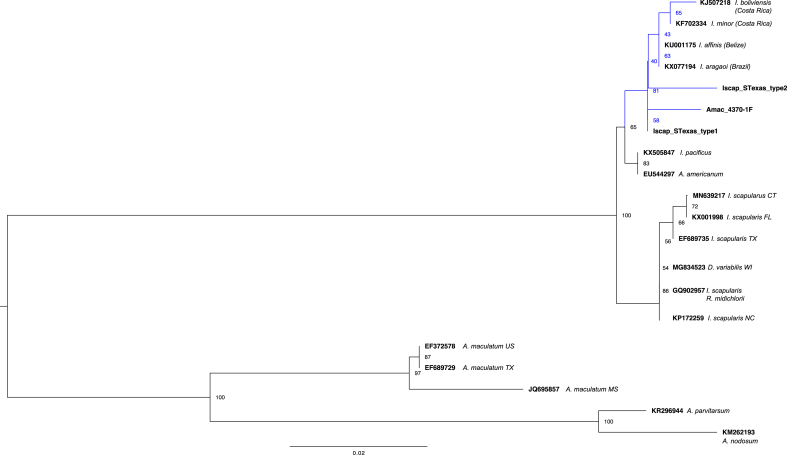
Phylogentic analysis of sca0 (*rompA*) sequences from putative *Rickettsia* sp. endosymbionts of *Amblyomma maculatum* and *Ixodes scapularis* ticks collected from white-tailed deer in southern Texas. This is a maximum-likelihood tree that is rooted at midpoint. Branch support was assessed with 10,000 replicates of UFBoot bootstrap replication, and bootstrap percentages are indicated at each branch point in the tree. Sequences from GenBank used in the comparative analysis were annotated as rickettsial endosymbionts. Accession numbers and tick species from which sequence was identified are included on the branch label.

All *I. scapularis* ticks were negative for *Borrelia* sp., *Anaplasmatacae* sp., and *Theileria-Babesia* sp. However, a diagnostic amplicon for *Rickettsia* sp. sca0 (*rompA*) was detected in all 37 *I. scapularis* ticks representing ticks from both LANWR and ESR. Sequence data obtained for each tick displayed 99–100% nucleotide sequence identity to rickettsial endosymbionts reported from ixodid ticks (e.g., GenBank KX077194). Comparison of the *I. scapularis* sequences identified two highly similar genotypes with five polymorphic nucleotide positions (Iscap_STexas_type1 and _type 2). These were used for phylogenetic analysis along with the *Am. maculatum* endosymbiont sequence (Amac_4370-1F), which was identical to Iscap_STexas_type1 and is represented in the phylogeny (Fig. 4).

## 4 Discussion

White-tailed deer are known reservoirs of tick-borne pathogens, including *E. chafeensis* (Lockhart et al., 1997), *Ehrlichia ewingii* (Yabsley et al., 2002), *Ehrlichia* spp. (Yabsley et al., 2008) and *Anaplasma* spp. (Dugan et al., 2006). It is unclear what role nilgai may have in this capacity. Documenting the various tick species parasitizing these hosts expands what we know of the tick fauna in southern Texas that share a landscape with the invasive cattle fever tick, which was found singly and in combination with between one to three different tick species in the LANWR (Table 1, Table 2). Screening these tick specimens further provides insight into parasites circulating within these ungulate populations in this unique region of Texas. The most common tick encountered on deer at the LANWR was the tropical horse tick, *A. nitens*. We follow Borges et al. (1998) in recognizing *Anocentor* as a genus distinct from *Dermacentor.* It is the vector of equine babesiosis and equine piroplasmosis (Pfeifer Barbosa et al., 1995; Schwint et al., 2008), and it is the most important ectoparasite of horses and donkeys in Latin America (Borges et al., 2000). This species is distributed throughout Latin America as far south as Argentina and on the islands of the Caribbean. It expanded into Florida in 1958 but is otherwise found in the USA only in southern Texas (Drummond et al., 1969; Scoles et al., 2011). It is a one-host tick, and aside from equines, has been reported as incidental on cattle and dogs. There are single records on deer in Mexico (Guzmán-Cornejo et al., 2011) and Costa Rica (Carreno et al., 2001), Nelson et al. (2017) reported them parasitizing deer in the US Virgin Islands, and Szabo et al. (2003) found them infesting marsh deer, *Blastocerus dichotomus* Illiger, in Brazil. Inasmuch as equids, cattle and dogs are not native to the New World, the finding of this tick in all stages and in great numbers on deer in southern Texas suggests that the latter are the native hosts. Additional surveys of cervid populations in countries throughout Latin America would better inform this host association.

*Amblyomma maculatum*, the Gulf Coast tick, was also collected from deer in this study at both the LANWR and ESR. It occurs across the southern USA from Virginia to California then south to Paraguay (Lado et al., 2018). As its name implies, this species is native to the Gulf coast of Louisiana and Texas, and only within 160 km of the coast, according to Bishopp and Hixson (1936). However, in the latter half of the twentieth century climate change and growth of the livestock industry resulted in an expansion into most of the southern USA (Paddock and Goddard, 2015; Sonenshine, 2018; Teel et al., 2010). *Amblyomma maculatum* is an important pest of cattle and dogs (Cooley and Kohls, 1944) and is another tick that prefers the ears as the bite site. A three-host tick, the larvae infest primarily birds (Teel et al., 1998). The native nymphal host is likely coyote (*Canis latrans* Say), and native adult host in the USA is presumably white-tailed deer as they have been reported on deer from essentially every state in their range (Nadolny and Gaff, 2018; Teel et al., 2010), including Texas (Samuel and Trainer, 1970). *Amblyomma maculatum* is the alternate reservoir of *Hepatozoon americanum*, the causative agent of wasting disease in dogs (Johnson et al., 2009). Experimentally they can transmit Panola Mountain Ehrlichiosis for which deer are the susceptible reservoir in nature (Loftis et al., 2016), and *Cowdria ruminatium*, the etiological agent of heartwater (Mahan et al., 2000), which has not yet been reported in the USA.

Deer are the typical host of adult black-legged ticks, *I. scapularis*, which is a three-host tick (Wilson et al., 1990). *Ixodes scapularis* is the primary vector of *Borrelia burgdorferi,* causative agent of Lyme disease, over the northeastern and mideastern U.S. (Keirans et al., 1996; Oliver et al., 1993). While the distribution map by Dennis et al. (1998) does not show it extending into southern Texas, Feria-Arroyo et al. (2014) confirm that the tick is present on white-tailed deer in this region, including in Cameron County, and in adjoining northern Mexico. *Ixodes scapularis* is also known to transmit *Babesia odocoilei*, a parasite originally described from white-tailed deer that has been detected in Texas herds (Waldrup et al., 1989, 1990).

While only four specimens were identified, *A. inornatum* was found on deer and a single nilgai from the LANWR. This species was originally described from southern Texas, but its range includes most of Mexico (Cooley and Kohls, 1944; Guzmán-Cornejo et al., 2011). A 3-host tick, it has been found on rabbits, dogs, cattle, deer, and humans. The present is the first report on nilgai. In Mexico it has been found on both species of peccary (*Pecari tajacu* and *Tayassu pecari*), so the latter may be the typical native host. There are no records of pathogens transmitted by this tick, but Medlin et al. (2015) detected *Rickettsia* sp., *Borrelia* sp., and *Ehrlichia* sp. in questing *A. inornatum* from Webb County (Texas).

Rickettsial parasites have been detected in ixodid ticks sampled from white-tailed deer, including *A. phagocytophilum*, *R. parkeri*, *E. ewingii*, and rickettsial endosymbionts (Baldridge et al., 2009; Lockwood et al., 2018; Mays et al., 2016; Trout Fryxell et al., 2015). Of the tick specimens screened from our study region, *Anaplasma* sp. were identified from *A. nitens*, while *Anaplasma* sp., *Ehrlichia* sp., and *Rickettsia* sp. were identified in *A. maculatum* ticks. Only *Rickettsia* sp. were detected in *A. inornatum* and *I. scapularis* ticks. Identities were based on nucleotide sequence similarity of the PCR amplicons to the GenBank database, and >99% identity with 100% coverage was indicative of a significant match. Thus, an only 80% sequence identity of the *groEL* amplicons from 17 adult *A. nitens* and one *A. maculatum* to that of *A. bovis*/*A. phagocytophylum* suggests that a unique *Anaplasma* sp. is circulating in these white-tailed deer. Use of additional gene targets for further characterization is warranted, given the high proportion of *A. nitens* adults that tested positive (81/103, 79%). *Anaplasma platys*, identified from *A. nitens* larval samples, is a known pathogen of dogs and is transovarially and transstadially transmitted in *Rhipicephalus sanguineus* Latreille (Llanes and Rajeev, 2020; Snellgrove et al., 2020). *Anaplasma platys* has been previously reported from cervids, including a recent survey of white-tailed deer from the East Foundation's San Antonio Viejo Ranch (SAVR) in Starr and Jim Hogg counties of southern Texas (Li et al., 2016; Yu et al., 2020). Twenty percent of *A. maculatum* ticks, all from ESR, were positive for an *Ehrlichia* sp. Delta strain, recently reported from an *Amblyomma triste* Koch tick in Argentina (Cicuttin et al., 2020). As *A. platys* and the latter *Ehrlichia* sp. are of unknown pathogenicity in deer, these data serve as records of their circulation in this region.

Targeting the *Rickettsia* sp. sca0 (*rompA*) gene, no *Rickettsia* sp. were detected in *A. nitens* ticks. *Rickettsia parkeri*, the causative agent of *R. parkeri* rickettsiosis, was detected in 9% of the screened *A. maculatum* ticks, the species known to transmit this pathogen in the USA (Paddock et al., 2004). These ticks were from both the LANWR and ESR. *Rickettsia parkeri* was reported in *A. maculatum* larvae from small mammals in southeastern Texas (Castellanos et al., 2016), and Paddock et al. (2020) detected *R. parkeri* in adult *A. maculatum* collected from a road-kill mule deer (*Odocoileus hemionus*) in western Texas; the current study expands the records to positive *A. maculatum* ticks from cervids in southern Texas. Medlin et al. (2015) reported Candidatus *Rickettsia amblyommii* (now *Rickettsia amblyommatis*) from questing *A. inornatum* adults in Webb County, Texas, and our detection of *R. amblyommatis* from *A. inornatum* ticks on-host in this study indicates circulation in this region. *Rickettsia amblyommatis* is commonly detected in *A. americanum,* and it is considered the most widely distributed spotted fever group rickettsia in the Americas because of the diversity and range of ticks from which it has been detected (Karpathy et al., 2016). Rickettsial endosymbiont sca0 (*ompA*) sequences were amplified from one *Am. maculatum* and all 37 *I. scapularis* ticks screened. The sequences isolated in this study form a clade with public database entries annotated as rickettsial endosymbionts from *Ixodes affinis* Neumann*, Ixodes aragaoi* Fonsec, *Ixodes minor* Neumann, and *Ixodes boliviensis* Neumann and are a sister clade to representative endosymbiont sequences from *Ixodes pacificus* Cooley & Kohls and *A. americanum*. This clade is distinct from that housing rickettsial endosymbionts of *I. scapularis*, and it is on a separate lineage from rickettsial endosymbionts previously reported from *A. maculatum*, *Amblyomma parvitarsum* Neumann, and *Amblyomma nodosum* Neumann (Fig. 4). Detection of rickettsial endosymbionts is not uncommon in ticks collected from free-ranging deer and small mammals or arthropods in general (Castellanos et al., 2016; Trout Fryxell et al., 2015; Weinert et al., 2015), and their role in tick biology is still being explored (Hunter et al., 2015). The implication of co-detection of this endosymbiont with the *Ehrlichia* sp. Delta strain in *Am. maculatum* is unclear, as the pathogenicity of both rickettsial species is unknown in deer.

*Theileria cervi*, identified in a high proportion of *A. nitens* ticks but in neither *A. maculatum* nor *I. scapularis* in this study, is a hemoprotozoan parasite transmitted by *A. americanum* ticks to white-tailed deer (Kuttler et al., 1967). Robinson et al. (1967) surveyed deer from throughout Texas and documented that more than half of the 1600 deer sampled were positive for *Theileria* by blood smear analysis. Detected in both free-ranging and farmed cervids (Cauvin et al., 2019; Samuel and Trainer, 1970), *T. cervi* is considered somewhat non-pathogenic; however, its pathogenicity can be accentuated by the physiological state of the host, (i.e., poor nutrition, heavy tick burden, infection; (Robinson et al., 1967; Yabsley et al., 2005). Interestingly, while *A. americanum* is the demonstrated primary vector of *T. cervi* (Kuttler et al., 1967), none were collected during this surveillance effort. Samuel and Trainer (1972) described *A. americanum* from deer in San Patricio County (Texas), which is approx. 200 km north of the LANWR, and an *A. americanum* adult tick was recorded from a single nilgai host at ESR (DBT, unpublished); however, they are not typically reported from this area (Corn et al., 2016). This suggests *T. cervi* is maintained in the population by a different tick vector, and it appears to associate with a high prevalence of *A. nitens*. Other tick species, e.g., *R.* (*B.*) *microplus*, *A. mixtum* and *A. tenellum*, are encountered in this region as well (Corn et al., 2016; Olafson et al., 2018), but whether they harbor *T. cervi* is currently unknown.

Different genotypes of *Theileria* 18S SSU rRNA have been defined from various bovine and cervid hosts and geographic regions (Chae et al., 1998, 1999a), resulting in three different types (Type F, Type G, and ‘divergent’) from white-tailed deer, elk (*Cervus canadensis*), and mule deer in the USA (Cauvin et al., 2019; Chae et al., 1998, 1999b; Wood et al., 2013). A recent survey of deer on the SAVR (Starr and Jim Hogg Counties, Texas) indicated approx. 7% of 245 animals were positive for *T. cervi* (Yu et al., 2020). Analysis of the 16 publicly available *T. cervi* sequences from Yu et al. (2020) indicate a single genotype (Type F), while sequence data from *A. nitens* ticks in the current study indicate that all three cervid genotypes (Type F, G, and ‘divergent’) are circulating within this Cameron County (Texas) deer population (Fig. 3). Further, detection and confirmation of the *T. cervi* sequence from adult *A. nitens* collected from a single nilgai host suggests circulation of the piroplasm in this exotic ungulate species. Deer infested with *T. cervi* -positive *A. nitens* ticks were distributed across the units within LANWR and, using positive ticks as an indicator of parasite circulation, suggests a much higher prevalence (27/31, 87%) of *T. cervi* in deer of this region.

## 5 Conclusions

Southern Texas shares an ungulate fauna with northern Mexico: nilgai antelope, collared peccaries, white-tailed deer. As such, these ungulates can transport species of ticks across the international border, and with them associated pathogens. Among those of concern are bovine babesiosis, equine babesiosis, and anaplasmosis. Bovid and cervid hosts can sustain populations of various tick species in this region, thus the wildlife-livestock interface in southern Texas is of concern because of the potential for transmission of pathogens to livestock. Further, presence of the invasive cattle fever tick in this region is likely a result of dissemination by alternative hosts, such as deer and nilgai, impacting tick eradication efforts (Lohmeyer et al., 2018; Pound et al., 2010). Surveying ticks from deer and nilgai offer an opportunity to identify pathogens that may be circulating in these hosts. This study contributed records of the tick fauna parasitizing deer and nilgai and documented the circulation of sylvatic piroplasmas, anaplasmas and rickettsias in southern Texas.

## Declaration of competing interest

The authors declare that they have no competing interests.
