# Supplementary material for: Molecular screening for rickettsial bacteria and piroplasms in ixodid ticks surveyed from white-tailed deer (Odocoileus virginianus) and nilgai antelope (Boselaphus tragocamelus) in southern Texas
Source: Int J Parasitol Parasites Wildl. 2020 Nov 17;13:252–60. doi: 10.1016/j.ijppaw.2020.11.002 (PMC7691163; doi:10.1016/j.ijppaw.2020.11.002)
Supplement: The following is the supplementary data related to this article:Multimedia component 1 [file mmc1.docx]

**Table S1.** Molecular screening assays for detection of *Rickettsia* sp., *Anaplasmataceae* sp., *Theileria*-*Babesia* sp., and *Borrelia* sp. Oligonucleotide primer sequences, reaction conditions, and cycling parameters are summarized.

| Gene | Primer Sequence | Reaction Conditions | Cycling Parameters |
| --- | --- | --- | --- |
| *Rickettsia* sp.  *sca0* (*rompA*)  (Regnery et al. 1991) | Rr190.70P:  ATGGCGAATATTTCTCCAAAA  Rr190.602N:  AGTGCAGCATTCGCTCCCCCT | 2 mM MgCl_2_ 0.2 mM dNTP mix 0.2 mM each primer 0.5 U Platinum *Taq* | *Initial denaturation*: 95 °C, 2 mins  40 cycles of:  95 °C, 1 min; 55 °C, 1 min; 72 °C, 30 s  *Final Extension*: 72 °C, 2 min |
| *Anaplasmataceae* sp.  *groEL*  (Tabara et al. 2007) | Primary PCR  Ehr-gro607F:  GAAGATGCWGTWGGWTGTACKGC  Ehr-gro1294R  AGMGCTTCWCCTTCWACRTCYTC  Nested PCR  Ehr-gro 677F:  ATTACTCAGAGTGCTTCTCARTG  Ehr-gro1121R:  TGCATACCRTCAGTYTTTTCAAC | 1.25 mM MgCl_2_ 0.4 mM dNTP mix 0.5 mM each primer 1.25 U Platinum *Taq* | *Initial denaturation*: 95 °C, 2 mins  30 cycles (primary), 35 cycles (nested):  95 °C, 30 s; 57 °C, 20 s; 72 °C, 30 s  *Final Extension*: 72 °C, 1 min |
| *Theileria* sp.*/Babesia* sp.  18S rRNA SSU V4  (Oosthuizen et al. 2008, Ueti et al. 2015) | Primary PCR/clone sequencing  Nbab-1F:  AAGCCATGCATGTCTAAGTATAAGCTTTT  Nbab-1R:  CTTCTCCTTCCTTTAAGTGATAAGGTTCAC  Nested PCR/clone sequencing  RLBinner-F:  AATCCTGACACAGGGAGGTAGTGAC  RLBinner-R:  CTAAGAATTTCACCTCTGACAGT | 1.25 mM MgCl_2_ 0.2 mM dNTP mix 0.4 mM each primer 0.5 U Platinum *Taq* | *Initial denaturation*: 95 °C, 2 mins  (Primary)  35 cycles of:  95 °C, 30 s; 57 °C, 20 s; 72 °C, 30 s  (Nested)  35 cycles of:  95 °C, 30 s; 65 °C, 20 s; 72 °C, 30 s  *Final Extension*: 72 °C, 4 mins |
| *Borrelia* sp.  *flaB*  (Barbour et al. 1996) | flaLL:  ACATATTCAGATGCAGACAGAGGT  flaRL:  GCAATCATAGCCATTGCAGATTGT | 2 mM MgCl_2_ 0.2 mM dNTP mix 0.25 mM each primer 1 U Platinum *Taq* | *Initial denaturation*: 95 °C, 2 mins  30 cycles (primary), 35 cycles (nested):  95 °C, 1 min; 55 °C, 1 min; 72 °C, 30 s  *Final Extension*: 72 °C, 4 mins |

**Table S2.** Summary of *Anocenter nitens* ticks that tested positive for *Theileria cervi* by PCR targeting the 18S SSU rRNA gene. Life stage, ‘number positive/total’, and number of larvae per sample processed are noted. F: adult female, M: adult male, N: nymph, and L: larvae.

|  |  | ***Anocenter* *nitens*** | | | |  |
| --- | --- | --- | --- | --- | --- | --- |
| **Date Collected** | **Animal ID** | **F** | **M** | **N** | **L** | **Larvae per tube** |
| 12-7-2018 | W-1532 | 1/1 | 4/5 |  | 3/3 | 10/10/10 |
| 12-7-2018 | W-1533 |  |  |  | 1/1 | 15 |
| 12-7-2018 | W-1534 |  |  |  | 2/2 | 13/13 |
| 12-8-2018 | W-1536 | 1/2 |  |  | 2/3 | 12/11/11 |
| 12-8-2018 | W-1538 |  | 1/4 | 1/7 |  |  |
| 12-8-2018 | W-1539 |  |  |  | 16/16 | 5 x 16 tubes |
| 12-8-2018 | W-1540 | 4/28 | 20/29 |  |  |  |
| 12-9-2018 | W-1541 |  |  |  | 1/1 | 7 |
| 12-9-2018 | W-1542 | 0/5 | 0/9 | 0/1 | 0/1 | 2 |
| 12-9-2018 | W-1544 | 0/1 |  |  | 2/2 | 9/9 |
| 12-9-2018 | W-1545 |  |  |  | 2/2 | 11/10 |
| 12-9-2018 | W-1546 |  |  |  | 1/2 | 6 |
| 12-9-2018 | W-1547 | 3/12 | 2/13 | 0/1 |  |  |
| 12-9-2018 | W-431 |  | 3/7 | 8/38 | 1/2 | 10/10 |
| 12-9-2018 | W-432 |  | 1/6 | 0/1 | 5/5 | 10/10/10/8/8 |
| 12-9-2018 | W-1550 | 2/3 | 0/2 |  |  |  |
| 12-10-2018 | W-1701 | 0/1 | 0/1 |  |  |  |
| 12-10-2018 | W-1702 |  | 0/2 | 0/5 |  |  |
| 12-10-2018 | W-1704 | 1/6 | 4/12 |  |  |  |
| 12-11-2018 | W-1706 | 0/5 | 0/1 |  | 0/1 | 1 |
| 12-11-2018 | W-1707 | 2/3 | 14/23 | 1/4 | 0/2 | 11/11 |
| 12-11-2018 | W-1708 |  | 2/7 | 0/2 | 1/1 | 2 |
| 12-11-2018 | W-1709 |  |  | 11 | 1 | 3 |
| 12-14-2018 | W-1710 | 0/4 | 1/11 | 1/11 | 1/1 | 3 |
| 12-14-2018 | W-1711 |  | 2/3 | 0/7 | 0/1 | 7 |
| 12-14-2018 | W-1712 |  | 1/2 |  | 1/1 | 8 |
| 12-15-2018 | W-1714 |  |  | 0/13 | 1/1 | 1 |
| 12-15-2018 | W-1716 | 2/14 | 4/13 | 2/7 | 1/1 | 1 |
| 12-16-2018 | W-1717 |  |  | 0/2 | 1/2 | 13/12 |
| 12-16-2018 | W-1718 |  |  | 2/5 | 3/3 | 10/10/11 |
| 12-16-2018 | W-1719 |  |  |  | 1/1 | 15 |

**Supplemental References.**

**Barbour, A. G., G. O. Maupin, G. J. Teltow, C. J. Carter, and J. Piesman. 1996.** Identification of an uncultivable Borrelia species in the hard tick Amblyomma americanum: possible agent of a Lyme disease-like illness. J Infect Dis 173: 403-409.

**Oosthuizen, M. C., E. Zweygarth, N. E. Collins, M. Troskie, and B. L. Penzhorn. 2008.** Identification of a novel Babesia sp. from a sable antelope (Hippotragus niger Harris, 1838). J Clin Microbiol 46: 2247-2251.

**Regnery, R. L., C. L. Spruill, and B. D. Plikaytis. 1991.** Genotypic identification of rickettsiae and estimation of intraspecies sequence divergence for portions of two rickettsial genes. J Bacteriol 173: 1576-1589.

**Tabara, K., S. Arai, T. Kawabuchi, A. Itagaki, C. Ishihara, H. Satoh, N. Okabe, and M. Tsuji. 2007.** Molecular survey of Babesia microti, Ehrlichia species and Candidatus neoehrlichia mikurensis in wild rodents from Shimane Prefecture, Japan. Microbiol Immunol 51: 359-367.

**Ueti, M. W., P. U. Olafson, J. M. Freeman, W. C. Johnson, and G. A. Scoles. 2015.** A Virulent Babesia bovis Strain Failed to Infect White-Tailed Deer (Odocoileus virginianus). PLoS One 10: e0131018.
